# Evaluation of the Fresh Properties of Cement Pastes: Part I—Response Models Applied to Changes in the One-Variable-at-a-Time Method

**DOI:** 10.3390/ma16114139

**Published:** 2023-06-01

**Authors:** Lino Maia

**Affiliations:** 1 CONSTRUCT-LABEST, Faculty of Engineering (FEUP), University of Porto, Rua Dr. Roberto Frias, 4200-465 Porto, Portugal; linomaia@fe.up.pt; 2 Faculty of Exact Sciences and Engineering, University of Madeira, Campus da Penteada, 9020-105 Funchal, Portugal

**Keywords:** approach, cement paste, fresh properties, modelling, trial–error

## Abstract

The workability of cement-based materials is one of the features that makes these construction materials the most used worldwide. Measuring and understanding how cement-based constituent materials affect fresh properties depends on the experimental plans. The experimental plans deal with the constituent materials used, the tests carried out, and the run of experiments. Here, the fresh properties (workability) of cement-based pastes are evaluated based on the diameter in the mini-slump test and the time in the Marsh funnel test being measured. This overall study is composed of two parts. In Part I, tests were carried out on several cement-based paste compositions incorporating distinct constituent materials. The effects of the distinct constituent materials on the workability were analyzed. Furthermore, this work deals with an approach to the run of experiments. A typical run of experiments was applied, with basic sets of mixed compositions being studied by only changing one input parameter at a time. This approach used in Part I is faced with a more scientific approach applied in Part II of the work where, based on the design of experiments, multiple input parameters were changed at a time. This work showed that a basic run of experiments is quick and easy to apply and leads to results for simple analyses; conversely, it lacks information for advanced analyses and scientific conclusions. The tests carried out included studies on the effect on the workability caused by changes in the limestone filler content, the type of cement, the water-to-cement ratio, distinct superplasticizers, and shrinkage-reducing admixture.

## 1. Introduction

Cement-based materials are widely used because they are easy to understand. For instance, to produce a mortar for bed and head joints in masonry, essentially, sand, cement, and water are mixed in a trial–error method. Conversely, when high-performance properties are required without failure, high-tech knowledge based on scientific methodologies is vital. For instance, for high-tech cement-based materials, wherein the fresh properties are the crux of the matter (such as the ones with self-compacting properties or the ones optimized for 3D printing), the selection of the constituent materials and their proportions relies on scientific methods in order to make the correct choice.

Regarding the fresh state of a cement-based material, the most important rheological properties that characterize its workability are the yield stress and the plastic viscosity. The most precise and scientific approach to evaluating these two properties is by using a rheometer. However, rheometers are quite expensive, require specific skills, are difficult to operate, require specific conditions, and have reduced reproductivity, among other problems such as expulsion or fracture of the material slip flow [1,2,3]. Therefore, concrete engineers worldwide have used the slump cone test (the Abrams test in concrete)—an inexpensive and highly reproducible test [4]. Additionally, the V-funnel test for self-compacting concrete (or the reduced V-funnel test for mortars) or the Marsh funnel test for pastes are also inexpensive, well-known, and highly reproducible tests that are widely (especially in laboratory work) used to evaluate the fresh state.

The mini-slump test carried out on pastes is usually carried out using the equipment described in Ref. [5], with the output being the flow diameter. The Marsh funnel test on pastes is usually carried out using the equipment described in the standards EN445 or ASTM C939-10 (among others, Refs. [6,7,8,9,10]), with the output being the flow time. According to several studies [11,12,13,14,15], the flow diameter is more correlated with the yield stress, whereas the flow time is a combination of the yield stress and the plastic viscosity [10].

Thus, in the absence of a rheometer, when a large number of tests are required, or for a quick evaluation, the mini-slump and Marsh funnel tests are accepted as the tests that mostly characterize the workability properties of cement-based pastes [16], especially when the superplasticizer effect or saturation is being studied [6,7]. In this research, the workability properties of several pastes were assessed using these two tests.

Another concern that arises is the type of approach, i.e., the design of experiments (DOE) technique, to achieve scientific results. Typically, simple approaches are used where the researcher individually changes one or two parameters. These approaches, performing only a few tests, obtain results and allow for some understanding regarding the effect caused by the changed parameters. Habitually, with these approaches, the results are plotted in charts in Microsoft Excel, and linear regression (or fitting curves) trends are determined to make quick and direct comparisons between the input parameters. However, when a large characterization is required with many input variables to be tested, other approaches are more appropriate. Various DOE approaches include those based on statistics, such as the experiments carried out by Taguchi [17,18], factorial design [19,20,21,22], the response surface method [23,24,25], methods related to artificial neural networks [26,27], and support vector machines [28,29,30,31], among others. Therefore, the choice of an appropriate DOE performance is improved. Occasionally, the typical approach (also called the traditional approach in this paper) is chosen because it is very easy, and the primary outputs are comprehensible. On other occasions, a factorial design is chosen, with all of the input variables being changed at once, and with response models applied to the results to detect direct and cross effects due to changes in the input variables.

In Part I, the research focuses on evaluating distinct experimental approaches to assess the fresh properties of cement-based pastes. The present paper is Part I of this research and presents the findings regarding experimental programs implementing a traditional approach. In Part II, the experimental program is designed based on a statistical approach, i.e., a central composite design of experiments is used. The major goals of this paper (Part I) are as follows: (i) to identify the principal effects of each constituent material of cement pastes on workability, and (ii) to evaluate and think about the type of research methodology used, namely, the pros and cons of the traditional approach compared to an experimental program design based on a central composite design of experiments.

Therefore, for Part I, 92 mixture compositions were made, whereas 15 mixture compositions were produced for Part II. All studies were conducted using cement-based pastes. The tests carried out to evaluate the fresh properties were the mini-cone test and the Marsh funnel test. It should be noted that Part I and Part II are not comparable in terms of their results. Taken together, the results of Part I and Part II complement each other in order to improve the overall characterization of the effects of the constituent materials, in addition to allowing for an evaluation and comparison of distinct approaches.

## 2. Experimental Program

The experimental program presented is the result of several studies developed to understand and characterize the effects of different constituent materials on the fresh properties of cement-based pastes. These studies were carried out using well-defined procedures; however, the mixtures were not defined at the beginning of the experimental program. In fact, as noted below, some materials were barely used (three superplasticizers, one cement, and one SRA), but their results are presented because they are also a part of the experimental work of the typical methodology, and they contribute toward the overall aim of the study.

### 2.1. Materials

Three powders were used in this study:

(i). Commercial cement CEM I 42.5R (EN 197-1) produced by SECIL, Outão, Portugal, with 90.2% of clinker, 5.2% of gypsum, 4.5% of limestone filler, Blaine 3871 cm^2^/g, and a specific gravity of 3.11 g/cm^3^—this cement was used as reference one;
(ii). Commercial cement CEM I 52.5R (EN 197-1) produced by SECIL, Outão, Portugal, with 93.3% of clinker, 6.7% of gypsum, 0% limestone filler, Blaine 4803 cm^2^/g, and a specific gravity of 3.13 g/cm^3^;
(iii). Limestone filler with a specific gravity of 2.70 g/cm^3^.

Five commercial organic admixtures (EN 934-2) in the liquid state were used as follows: (i) four distinct third-generation superplasticizers herein named (A), (B), (C), and (D), with 25.5%, 18.0%, 40%, and 30% of solid content, and with 1.02, 1.05, 1.08, and 1.06 g/cm^3^ of specific gravity, respectively, and (ii) a shrinkage-reducing admixture (SRA) in a liquid state with 1020 g/cm^3^ (there was no other information, especially regarding the solid content).

### 2.2. Mixture Compositions

In total, 92 mixtures were made. These mixtures can be divided into two large groups according to their powder content: Group (I)—mixtures 1 to 54 made in batches with 160 g of powder; and Group (II)—mixtures 55 to 92 made in batches of 1.40 L with 2693.7 g of powder. All the mixture compositions are displayed in Table 1, Table 2 and Table 3. In Table 1, the 47 mixtures made without organic admixtures are presented; in Table 2, the 41 mixtures made with superplasticizer but without SRA are presented; and in Table 3, the four mixtures made with SRA are presented.

The mixtures in Table 1 refer to seven sets of mixtures arranged according to the changes from mix to mix—these seven sets were used for the same study. Mixtures 1–7 refer to a set used to evaluate the effect of the water content and/or the water-to-cement ratio (this set was used as reference one). Mixtures 8–14 refer to a set in which 15% of the cement was replaced by limestone filler. Mixtures 15–21 refer to a set in which 30% of the cement was replaced by limestone filler. Mixtures 22–30 refer to a set in which 45% of the cement was replaced by limestone filler. Mixtures 31–37 refer to a set in which all the cement was replaced by limestone filler. Mixtures 38–42 refer to a set in which cement CEM I 42.5R was replaced by cement CEM I 52.5R. Mixtures 43–47 refer to a set in which 20% of the cement CEM I 42.5R was replaced by cement CEM I 52.5R.

The mixtures in Table 2 refer to three sets of mixtures arranged according to the changes from mix to mix. Mixtures 48–54 refer to a set used to evaluate the effect of two superplasticizers. Mixtures 55–70 refer to a set used to evaluate the effect of another superplasticizer in two different water-to-cement ratios. Mixtures 71–88 refer to a set used to evaluate the effect of two superplasticizers in compositions with limestone filler. The mixtures in Table 3 refer to a set in which the effect of an SRA was evaluated. Mixtures 82, A, B, C, and D refer to a set used to evaluate how the incorporation of SRA affects fresh properties.

### 2.3. Mixing and Testing

The mixtures of Group (I) were made according to the following procedure: (i) cement and then the limestone filler (if applicable) were weighed in a plastic cup; (ii) the water was weighed in another plastic cup; (iii) if applicable, the superplasticizer was weighed in a syringe; (iv) the water was poured over the powder; (v) the superplasticizer was poured over the other compounds; (vi) all the compounds were mixed with a paddle at 2000 r.p.m. for two minutes.

To evaluate the deformability of the pastes through flowability, the mini-slump test was carried out immediately after mixing, and immediately after that, this test was repeated once more without cleaning the equipment (mini-cone). Four readings were recorded by measuring two orthogonal distances in each test.

The mixtures of Group (II) were made with mixers typically used in tests of standard pastes of European Norms 196. However, the procedure was adapted as follows: (i) all the materials were previously weighed; (ii) powders and ~80% of water were poured into the mixing container; (iii) mixing was performed for 1 min at low speed; (iv) mixing was stopped and the remaining water and superplasticizer were poured over the paste; (v) mixing took place for 1 min at low speed; (vi) mixing was stopped for 30 s and the SRA (if applicable) was poured; (vii) mixing was performed for 2 min at low speed; (viii) mixing was stopped for 15 s; (ix) mixing continued for 30 s at high speed.

Immediately after mixing, the mini-slump tests were carried out similarly to the mixtures of Group (I). Then, a Marsh funnel test was performed to evaluate the viscosity of pastes. After these tests, the material was placed in a plastic container, and the mini-slump test and Marsh funnel test were carried out once more 60 min after the start of mixing. It should be noted that for some mixtures, not all the tests described here were carried out.

**Figure 1:**
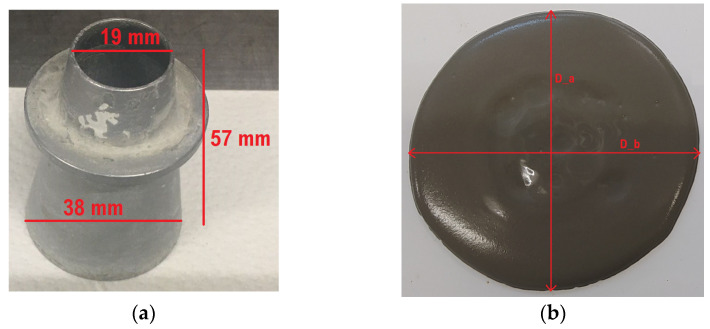
(**a**) Mini cone device; (**b**) flow diameters.

The mini-slump test setup used in this study was a downscaled Abrams cone geometry [5] with a 19 mm top diameter, 38 mm bottom diameter, and 57 mm height. Each test was performed using a stainless steel mini-slump cone on a flat sheet of stainless steel. Figure 1 presents the device and flow of the paste.

The Marsh funnel (EN445) test was the one typically adapted for cement-based pastes; i.e., 1000 mL of paste was poured into the Marsh funnel, and then the time for a measuring cup to reach 500 mL was recorded.

## 3. Results and Discussion

### 3.1. Experimental Results

The experimental results are fully reported in Table 4 and Table 5. In Table 4, ‘D1a’ and ‘D1b’ are the two orthogonal measurements of the first mini-slump test, ‘D2a’ and ‘D2b’ are the measurements for the second test, and ‘D-flow’ is the average of all measurements taken. In this table, the ‘a’ (slope), ‘b’ (ordinate at the origin), and the ‘R^2′^ of the linear regression of the corresponding set of mixtures are also shown. In Table 5, ‘D0’1a’, ‘D0’1b’, ‘D0’2a’, ‘D0’2b’, and ‘D0′ refer to measurements of the mini-slump test taken immediately after mixing and corresponding to the D1a’, ‘D1b’, ‘D2a’, ‘D2b’, and ‘D-flow’ described above, respectively. The measurements of ‘D60’1a’, ‘D60’1b’, ‘D60’2a’, ‘D60’2b’, and ‘D60′ are similar but taken 60 min after the start of mixing. Furthermore, ‘t0′ and ‘t60′ are the time of Marsh funnel test taken immediately after mixing and 60 min after the start of mixing, respectively.

### 3.2. Analysis of Results and Discussion

#### 3.2.1. Flowability of Mixtures with Different Limestone Filler Content, Different Types of Cement, and without Organic Admixtures

**Figure 2:**
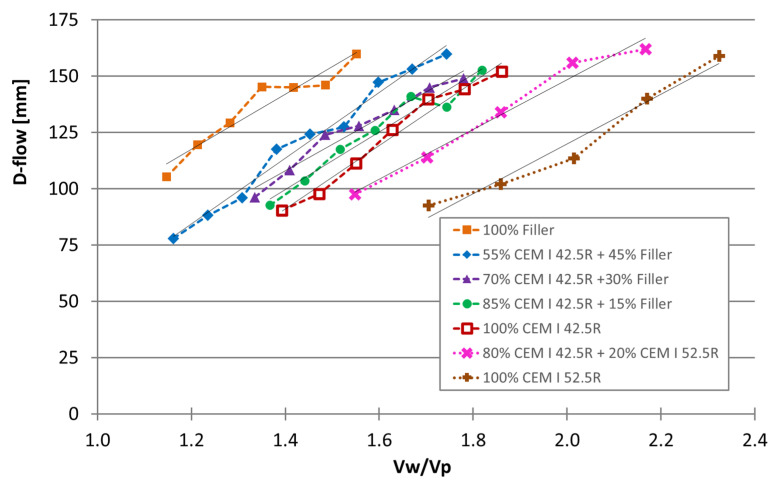
Flowability of mixtures 1 to 47, wherein sets with different limestone filler contents and different types of cement were studied.

The results of the flowability immediately after mixing the composition without organic admixtures (mixture compositions of Table 1) are graphically shown in Figure 2. The lines of the linear regressions are also displayed, with the values of the slope, ordinate at the origin, and R^2^ listed in Table 4. The set of mixtures named ‘100%CEM I 42.5R’ was used as a reference to compare with the other sets. As expected, and in agreement with the literature [32,33,34,35], one observes that as much as the limestone filler replaces cement, flowability increases for the same water-to-powder ratio. Furthermore, when cement CEM I 52.5R replaced CEM I 42.5R, a strong increase in the water was required to reach the same flowability. This effect might be explained by the cement fineness because finer cement with higher water content is required to reach the same flowability [21,36,37].

In fact, it was observed that the replacement of cement CEM I 42.5R by CEM I 52.5R had a stronger effect on flowability than when it was replaced by limestone filler, the effects of which were inverse. This can be observed not only when cement CEM I 42.5R is completely replaced but also when it was partially replaced. This is probably due to the particle shape and size distribution of the cement with a higher specific surface area, resulting in lower flowability. Unfortunately, neither the particle shape nor the particle size distribution information was available for the powders. However, it is known that cement CEM I 42.5R typically has lower granulometry and higher specific surface area (cm^2^/g) than the limestone filler, but higher granulometry and lower specific surface area than cement CEM I 52.5R.

#### 3.2.2. Effect of Superplasticizers on the Workability Parameters

**Figure 3:**
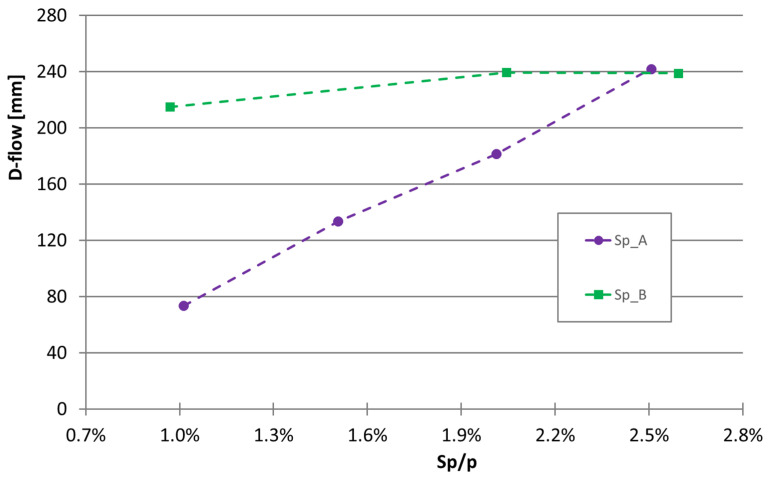
Effect of the superplasticizers A and B (mixtures 48 to 54) on the flowability of mixtures with w/c = 0.285 and without limestone filler.

As previously reported, four superplasticizers were used. However, the first two superplasticizers were used in a simple study wherein the flowability was checked through a mini-slump test in mixtures within Group (I); i.e., mixtures made with 160 g of cement. Figure 3 presents the graphical results reported in Table 5 for mixtures 48 to 54. As can be seen in Figure 3, over the range of superplasticizer contents used, the effect on flowability is markedly different. While superplasticizer A has a markedly extensive effect close to linear (the higher the superplasticizer content, the higher the deformability), the effect of superplasticizer B is much smaller for the contents evaluated. It appears that superplasticizer B already has its maximum effect to improve fluidity at Sp/p = 1%.

**Figure 4:**
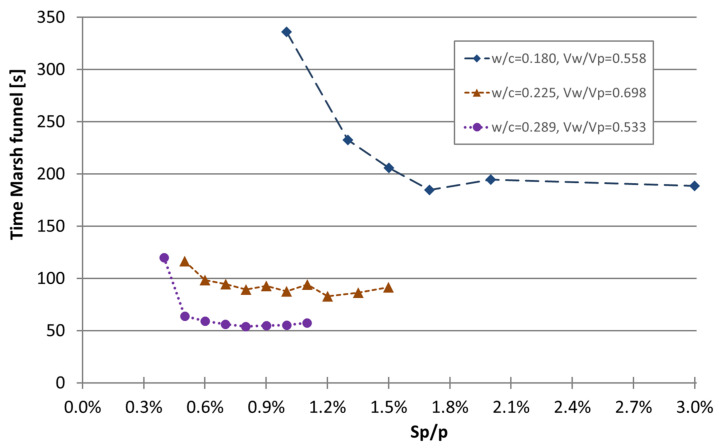
Effect of the superplasticizer C and the water-to-cement ratio (mixtures 55 to 78) on viscosity evaluated through the time of the Marsh funnel test.

Based on the previous findings, the author expanded the study of the effect of the superplasticizer on fresh properties by carrying out another set of mixtures (mixtures 55 to 70 without limestone filler and mixtures 71 to 78 with limestone filler) with low w/c ratios, where another stronger superplasticizer (named Superplasticizer C) was used, and where its effect on the viscosity was evaluated using the Marsh funnel test. The test results of the Marsh funnel test are graphically shown in Figure 4. As can be observed with Superplasticizer C, the effect on the funnel Marsh time is markedly different when the w/c ratio changes from 0.180 to 0.225 (mixtures without limestone filler). In general, by reducing the water-to-cement ratio by 20% from 0.225 to 0.180, the minimum time of the Marsh funnel doubled and required more than twice the Sp/p to reach the minimum time. Furthermore, when analyzing the effect of the superplasticizer on the set of mixtures with w/c = 0.289 but with 40% of the powder as limestone filler, the viscosity decreased even more. This finding is particularly important because this set of mixtures, although with a higher water-to-cement ratio, had the lowest water content and lower water-to-powder ratio in volume (Vw/Vp = 0.533 compared to Vw/Vp = 0.698 and Vw/Vp = 0.558). Therefore, one may conclude that the water-to-cement ratio plays an important role in the viscosity and is even stronger than the water content.

**Figure 5:**
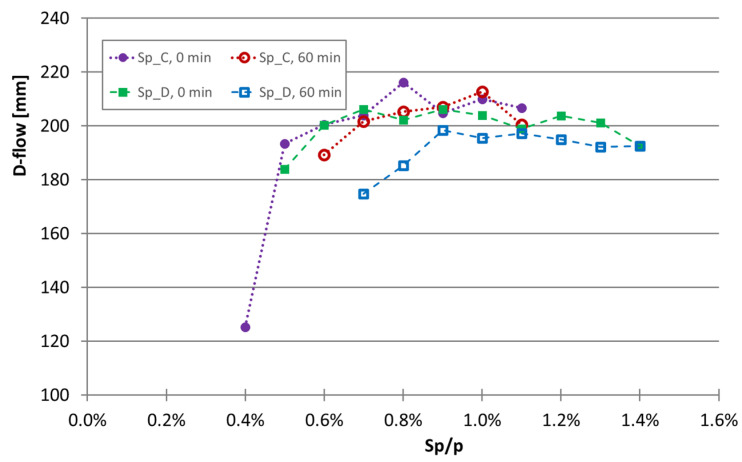
Effect of the superplasticizers C and D on the flowability of mixtures immediately after mixing and 60 min after the start of mixing in mixtures with limestone filler.

**Figure 6:**
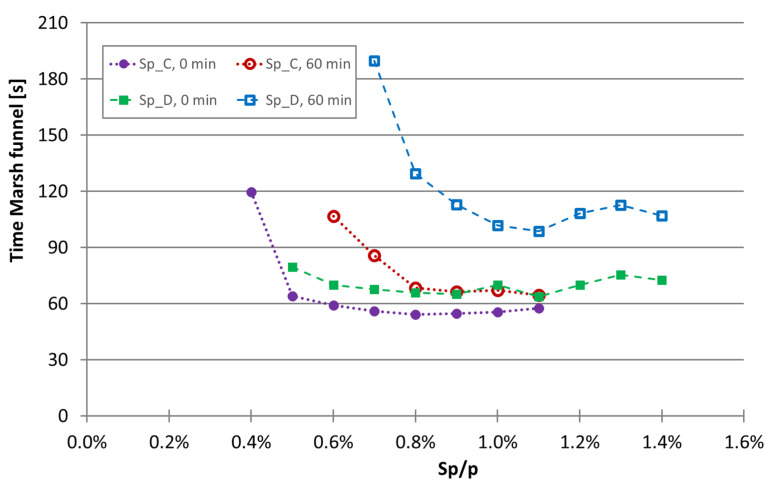
Effect of the superplasticizers C and D on the viscosity immediately after mixing and 60 min after the start of mixing assessed through the time of the Marsh funnel test in mixtures with limestone filler.

The loss of workability over time in the experimental program was also evaluated. Moreover, two superplasticizers were tested (mixtures 71 to 88) to identify modifications. The loss of deformability of the superplasticizers C and D is shown graphically in Figure 5 from immediately after mixing to up to 60 min after the start of mixing. Similarly, the increase in the viscosity is shown in Figure 6.

By comparing the two superplasticizers, it is concluded that in terms of deformability (assessed through the flow in the mini-slump test), changes are not markedly different. In fact, some loss of flowability was observed from immediately after mixing to 60 min after mixing, with the loss flowability being greater for superplasticizer D. However, when the increase in viscosity was analyzed by the increase in the time of the Marsh funnel, a marked difference was observed between testing immediately after mixing and 60 min after mixing. This viscosity increase occurred in both superplasticizers, but it is more pronounced with superplasticizer D, even at a higher superplasticizer content. Therefore, it can be concluded that superplasticizer C not only has a stronger effect than superplasticizer D but also shows less workability loss up to 60 min after the start of mixing.

#### 3.2.3. Experimental Results with the Shrinkage-Reducing Admixture

One of the questions faced mainly by researchers (because they must be scientifically accurate) when admixtures are introduced in a cement-based composition is how to introduce them. Questions such as the following arise: (i) Is it to be assumed that as the total volume increases, the per cubic meter of all other constituent materials is reduced? (ii) When the admixture is in the liquid state, must the same volume of water be removed? (iii) How can this be introduced without altering the workability or the compressive strength? Here, the incorporation of SRA was tested in three different ways, and the effect on workability was analyzed.

**Figure 7:**
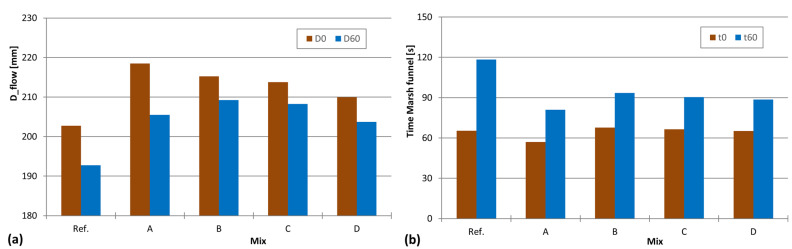
Changes in workability of mixtures with SRA introduced by different replacements: (**a**) deformability evaluated through the mini-slump test, and (**b**) viscosity evaluated through the time in the Marsh funnel.

For this purpose, the author of this study took mixture 82 as the reference mix. Then, the following changes were made: Mixture A—20 g of SRA was added to the batch without removing any other component (the author considered that the SRA will occupy the pores of the material and/or assumed that the total volume of the mixture will increase); Mixture B—20 g of SRA was added to the batch by directly replacing 20 g of water (we considered that the SRA will behave exactly like water); Mixture C—20 g of SRA was added to the batch by replacing 10 g of water (50% replacement, which is an intermediary stage between Mixtures A and B); Mixture D—40 g of SRA was added to the batch by directly replacing 20 g of water (similar to Mixture C but with doubled SRA). The effect of the SRA on workability is presented in Figure 7.

By analyzing Figure 7, it can be concluded that regardless of the way in which the SRA is introduced, it always results in an increase in workability. As shown in Figure 7a, there is a marked increase in the flowability, and Figure 7b shows that the time of the Marsh funnel decreased. Furthermore, it is noted that the loss of workability after 60 min, especially the increase in viscosity (evaluated by the time of the Marsh funnel), is less pronounced for mixtures with SRA. However, no clear trends were found regarding how SRA should be introduced. Looking at the results of Mixture A, it is verified that simply adding the SRA to the batch without replacing any amount of water results in the greatest increase in workability; therefore, the author concludes that this form should be excluded. It should be noted that the compressive strength has not been evaluated; this probably also results in a reduction in the compressive strength as it increases the fluid part of the mixture. Among Mixtures B, C, and D all the results were quite similar; therefore, for simplification, the author suggests that the same mass content of water should be replaced when SRA is introduced.

## 4. Evaluation of the Approach Used

This Section aims to evaluate the applied approach and compare it with an alternative approach. In this paper, the typical approach, wherein the input parameters are changed individually, was applied to verify their effect on the workability of cement-based pastes. A visualization of the advantages and disadvantages of this approach, when compared to the approach used in Part II is, described below.

Typical approach—Advantages:

- Basic studies allow the interpretation of the main effects of the input parameter on the properties. Its effect is easier to test and verify for different materials. Here, a different cement, the effect of the SRA, and the effect of different superplasticizers were checked.
- In simple experimental plans with few mixtures, some trends were recognized.
- The experimental plans can easily be adjusted during the planning.
- Trail–error experiments may be enough to answer some doubts. In this paper, the SRA study was performed just to clarify a basic doubt. Likewise, superplasticizers A and B were checked for single effects only.

Typical approach—Disadvantages:

- Studies that are usually carried out under minor and basic sets are easy to analyze and interpret, and therefore, do not allow applying advanced response models that are typically described with linear regressions and R^2^.
- Studies are not wide-ranging experiments; therefore, the data are not usually published because their scientific interpretation lacks support. For example, in this study, the results of the SRA were easy and quick to obtain, but they were scientifically not interesting.
- When several properties need to be evaluated, the experimental program increases considerably, and the results and comparisons between the parameters are difficult to interpret.
- Secondary and cross effects are difficult to detect as no advanced response models are obtained.
- The results have not been deeply studied because they frequently lack a scientific approach—for instance, in this work, only a few mixtures were performed in the SRA study.

## 5. Conclusions

The workability of cement-based pastes using distinct experimental approaches was characterized in this work. This paper (Part I) mainly focuses on several basic studies, wherein sets of simple experimental plans were adjusted to identify primary effects. A different approach (more scientific, based on DOE with a central composite design) is proposed in Part II. In this study, 92 mixture compositions were made, and from the overall research, the following conclusions have been drawn:

- Basic approaches, which consist of changing a single parameter at a time, are very useful for quick studies whose objective is to recognize the main trends.
- These basic approaches allow the detection of the main effects of a single input variable with few trials. However, when multiple input variables are studied, the number of trials increases markedly, especially when the interactions between variables are studied.
- For multiple input variables with interaction effects, other DOE approaches should be considered, especially when deep analysis or models are required.
- The primary effects of the different constituent materials of cement pastes on fresh properties were characterized in this study using a traditional approach of changing each parameter separately. At the same time, the type of research methodology applied was studied, namely, the advantages and disadvantages when compared with an experimental program based on a factorial design.
- The replacement of cement by limestone filler increased the flowability.
- The efficiency of superplasticizers in terms of flowability and time of Marsh funnel is markedly affected not only by the type of superplasticizer but also by the water–cement ratio.
- The SRA used improved workability, but no clear trend was found for the introduction of the SRA.

## Figures and Tables

**Table 1:** Mixture compositions with limestone filler [g].

#	Mixture	CEM I 42.5R	CEM I 52.5R	Filler	Powder	Water	w/c	w/p	Vw/Vp
1	100%C, 0%F-1	160.20	-	-	160.20	71.97	0.45	0.45	1.39
2	100%C, 0%F-2	160.00	-	-	160.00	75.99	0.48	0.48	1.47
3	100%C, 0%F-3	160.01	-	-	160.01	80.05	0.50	0.50	1.55
4	100%C, 0%F-4	160.01	-	-	160.01	84.02	0.53	0.53	1.63
5	100%C, 0%F-5	160.08	-	-	160.08	88.01	0.55	0.55	1.70
6	100%C, 0%F-6	160.05	-	-	160.05	92.00	0.58	0.58	1.78
7	100%C, 0%F-7	160.02	-	-	160.02	96.04	0.60	0.60	1.86
8	85%C, 15%F-1	136.00	-	24.01	160.01	72.11	0.53	0.45	1.37
9	85%C, 15%F-2	136.03	-	24.00	160.03	76.02	0.56	0.48	1.44
10	85%C, 15%F-3	136.02	-	24.01	160.02	79.98	0.59	0.50	1.52
11	85%C, 15%F-4	136.00	-	24.01	160.01	83.94	0.62	0.53	1.59
12	85%C, 15%F-5	136.00	-	24.01	160.01	87.98	0.65	0.55	1.67
13	85%C, 15%F-6	136.01	-	24.09	160.09	92.03	0.68	0.58	1.74
14	85%C, 15%F-7	136.04	-	24.04	160.08	96.04	0.71	0.60	1.82
15	70%C, 30%F-1	112.01	-	48.02	160.03	71.98	0.64	0.45	1.34
16	70%C, 30%F-2	112.03	-	48.04	160.07	76.00	0.68	0.48	1.41
17	70%C, 30%F-3	112.02	-	48.02	160.04	80.07	0.72	0.50	1.49
18	70%C, 30%F-4	112.05	-	48.06	160.11	83.98	0.75	0.53	1.56
19	70%C, 30%F-5	112.01	-	48.01	160.03	88.01	0.79	0.55	1.63
20	70%C, 30%F-6	112.01	-	48.00	160.02	92.01	0.82	0.58	1.71
21	70%C, 30%F-7	112.02	-	48.00	160.02	95.98	0.86	0.60	1.78
22	55%C, 45%F-1	88.03	-	72.05	160.07	64.02	0.73	0.40	1.16
23	55%C, 45%F-2	88.01	-	72.03	160.05	68.01	0.77	0.43	1.24
24	55%C, 45%F-3	88.03	-	72.01	160.04	72.02	0.82	0.45	1.31
25	55%C, 45%F-4	88.01	-	72.01	160.03	76.03	0.86	0.48	1.38
26	55%C, 45%F-5	88.02	-	72.02	160.03	79.99	0.91	0.50	1.45
27	55%C, 45%F-6	88.01	-	72.04	160.05	83.99	0.95	0.53	1.53
28	55%C, 45%F-7	88.01	-	72.03	160.04	88.02	1.00	0.55	1.60
29	55%C, 45%F-8	88.07	-	72.01	160.08	91.99	1.04	0.58	1.67
30	55%C, 45%F-9	88.01	-	72.02	160.03	96.00	1.09	0.60	1.74
31	0%C, 100%F-1	-	-	160.06	160.06	68.00	-	0.43	1.15
32	0%C, 100%F-2	-	-	160.10	160.10	71.91	-	0.45	1.21
33	0%C, 100%F-3	-	-	160.00	160.00	75.98	-	0.48	1.28
34	0%C, 100%F-4	-	-	160.09	160.09	80.05	-	0.50	1.35
35	0%C, 100%F-5	-	-	160.05	160.05	84.00	-	0.53	1.42
36	0%C, 100%F-6	-	-	160.01	160.01	88.06	-	0.55	1.49
37	0%C, 100%F-7	-	-	160.02	160.02	92.00	-	0.58	1.55
38	100%C+, 0%F-1	-	160.00	-	160.00	87.99	0.55	0.55	1.71
39	100%C+, 0%F-2	-	160.01	-	160.01	95.98	0.60	0.60	1.86
40	100%C+, 0%F-3	-	160.00	-	160.00	103.99	0.65	0.65	2.02
41	100%C+, 0%F-4	-	160.01	-	160.01	112.01	0.70	0.70	2.17
42	100%C+, 0%F-5	-	160.01	-	160.01	119.97	0.75	0.75	2.32
43	80%C, 20%C+, 0%F-1	128.04	32.07	-	160.11	79.95	0.50	0.50	1.55
44	80%C, 20%C+, 0%F-2	128.07	32.05	-	160.11	87.91	0.69	0.55	1.70
45	80%C, 20%C+, 0%F-3	128.01	32.02	-	160.04	95.96	0.75	0.60	1.86
46	80%C, 20%C+, 0%F-4	128.00	32.04	-	160.05	103.88	0.81	0.65	2.01
47	80%C, 20%C+, 0%F-5	128.01	32.06	-	160.06	111.89	0.87	0.70	2.17

**Table 2:** Mixture compositions with superplasticizer [g].

#	Mixture	Cement	Filler	Powder	Superplasticizer	Sp/p	Water Added	Effective Water	w/c	Vw/Vp
48	100%C, SpA1.0%	160.00	-	160.0	1.62(A)	1.0%	44.39	45.60	0.285	0.884
49	100%C, SpA1.5%	160.00	-	160.0	2.41(A)	1.5%	43.80	45.60	0.285	0.884
50	100%C, SpA2.0%	160.00	-	160.0	3.22(A)	2.0%	43.20	45.60	0.285	0.884
51	100%C, SpA2.5%	160.00	-	160.0	4.01(A)	2.5%	42.61	45.60	0.285	0.884
52	100%C, SpB1.0%	160.00	-	160.0	1.55(B)	1.0%	44.33	45.60	0.285	0.884
53	100%C, SpB2.0%	160.00	-	160.0	3.27(B)	2.0%	42.92	45.60	0.285	0.884
54	100%C, SpB2.6%	160.00	-	160.0	4.15(B)	2.6%	42.20	45.60	0.285	0.884
55	w/c.18, SpC1.0%	2783.0	-	2783.0	27.85(C)	1.0%	484.23	500.94	0.180	0.558
56	w/c.18, SpC1.5%	2783.0	-	2783.0	41.77(C)	1.5%	475.88	500.94	0.180	0.558
57	w/c.18, SpC2.0%	2783.0	-	2783.0	55.68(C)	2.0%	467.53	500.94	0.180	0.558
58	w/c.18, SpC3.0%	2783.0	-	2783.0	83.51(C)	3.0%	450.83	500.94	0.180	0.558
59	w/c.18, SpC1.3%	2783.0	-	2783.0	36.19(C)	1.3%	479.23	500.94	0.180	0.558
60	w/c.18, SpC1.7%	2783.0	-	2783.0	47.33(C)	1.7%	472.54	500.94	0.180	0.558
61	w/c.225, SpC0.5%	2554.0	-	2554.0	12.78(C)	0.5%	566.98	574.65	0.225	0.698
62	w/c.225, SpC0.8%	2554.0	-	2554.0	20.45(C)	0.8%	562.38	574.65	0.225	0.698
63	w/c.225, SpC1.2%	2554.0	-	2554.0	30.67(C)	1.2%	556.25	574.65	0.225	0.698
64	w/c.225, SpC1.5%	2554.0	-	2554.0	38.33(C)	1.5%	551.65	574.65	0.225	0.698
65	w/c.225, SpC1.0%	2554.0	-	2554.0	25.57(C)	1.0%	559.31	574.65	0.225	0.698
66	w/c.225, SpC0.6%	2554.0	-	2554.0	15.33(C)	0.6%	565.45	574.65	0.225	0.698
67	w/c.225, SpC0.7%	2554.0	-	2554.0	17.91(C)	0.7%	563.90	574.65	0.225	0.698
68	w/c.225, SpC0.9%	2554.0	-	2554.0	23.01(C)	0.9%	560.84	574.65	0.225	0.698
69	w/c.225, SpC1.1%	2554.0	-	2554.0	28.14(C)	1.1%	557.77	574.65	0.225	0.698
70	w/c.225, SpC1.35%	2554.0	-	2554.0	34.49(C)	1.35%	553.96	574.65	0.225	0.698
71	63%C, 37%F, SpC0.4%	1693.7	1000.0	2693.7	10.87(C)	0.4%	482.36	488.88	0.289	0.533
72	63%C, 37%F, SpC0.5%	1693.7	1000.0	2693.7	13.49(C)	0.5%	480.79	488.88	0.289	0.533
73	63%C, 37%F, SpC0.6%	1693.7	1000.0	2693.7	16.2(C)	0.6%	479.16	488.88	0.289	0.533
74	63%C, 37%F, SpC0.7%	1693.7	1000.0	2693.7	18.87(C)	0.7%	477.56	488.88	0.289	0.533
75	63%C, 37%F, SpC0.8%	1693.7	1000.0	2693.7	21.55(C)	0.8%	475.95	488.88	0.289	0.533
76	63%C, 37%F, SpC0.9%	1693.7	1000.0	2693.7	24.25(C)	0.9%	474.33	488.88	0.289	0.533
77	63%C, 37%F, SpC1.0%	1693.7	1000.0	2693.7	27.07(C)	1.0%	472.64	488.88	0.289	0.533
78	63%C, 37%F, SpC1.1%	1693.7	1000.0	2693.7	29.64(C)	1.1%	471.10	488.88	0.289	0.533
79	63%C, 37%F, SpD0.5%	1693.7	1000.0	2693.7	13.47(D)	0.5%	479.45	488.88	0.289	0.533
80	63%C, 37%F, SpD0.6%	1693.7	1000.0	2693.7	16.17(D)	0.6%	477.56	488.88	0.289	0.533
81	63%C, 37%F, SpD0.7%	1693.7	1000.0	2693.7	18.87(D)	0.7%	475.67	488.88	0.289	0.533
82	63%C, 37%F, SpD0.8%	1693.7	1000.0	2693.7	21.73(D)	0.8%	473.67	488.88	0.289	0.533
83	63%C, 37%F, SpD0.9%	1693.7	1000.0	2693.7	24.27(D)	0.9%	471.89	488.88	0.289	0.533
84	63%C, 37%F, SpD01.0%	1693.7	1000.0	2693.7	26.96(D)	1.0%	470.01	488.88	0.289	0.533
85	63%C, 37%F, SpD01.1%	1693.7	1000.0	2693.7	29.65(D)	1.1%	468.13	488.88	0.289	0.533
86	63%C, 37%F, SpD01.2%	1693.7	1000.0	2693.7	32.36(D)	1.2%	466.23	488.88	0.289	0.533
87	63%C, 37%F, SpD01.3%	1693.7	1000.0	2693.7	35.16(D)	1.3%	464.27	488.88	0.289	0.533
88	63%C, 37%F, SpD01.4%	1693.7	1000.0	2693.7	37.74(D)	1.4%	462.46	488.88	0.289	0.533

Note: all these mixtures were made with CEM I 42.5 R; four distinct superplasticizers named ‘A’, ‘B’, ‘C’, and ‘D’ were used.

**Table 3:** Mixture compositions with shrinkage-reducing admixture [g].

#	Mixture	Cement	Filler	Sp_D	SRA	Water Added	Replacing Water	Comment
82	Ref.	1693.7	1000	21.73	-	473.67	-	Mixture reference
A	SRA20, wr0%	1693.7	1000	21.73	20.00	473.67	0%	SRA was added without any reduction in water
B	SRA20, wr100%	1693.7	1000	21.73	20.00	453.67	100%	SRA was added with the corresponding mass of water being removed
C	SRA20, wr50%	1693.7	1000	21.73	20.00	463.67	50%	SRA was added with 50% of the corresponding mass of water being removed
D	SRA40, wr50%	1693.7	1000	21.73	40.00	453.67	50%	SRA was added with 50% of the corresponding mass of water being removed

**Table 4:** Results from the mixtures with limestone filler.

#	Mixture	D1a	D1b	D2a	D2b	D-Flow	Linear Regression
1	100%C, 0%F-1	90	90	91	91	90.5	
2	100%C, 0%F-2	95	95	100	101	97.8	
3	100%C, 0%F-3	109	110	112	114	111.3	a = 0.006961
4	100%C, 0%F-4	123	123	129	130	126.3	b = 0.770234
5	100%C, 0%F-5	139	139	140	141	139.8	R^2^ = 0.978597
6	100%C, 0%F-6	143	143	145	146	144.3	
7	100%C, 0%F-7	151	151	153	153	152.0	
8	70%C, 30%F-1	93	94	99	99	96.3	
9	70%C, 30%F-2	105	105	111	112	108.3	
10	70%C, 30%F-3	119	119	129	129	124.0	a = 0.008278
11	70%C, 30%F-4	125	126	130	131	128.0	b = 0.510641
12	70%C, 30%F-5	135	135	135	135	135.0	R^2^ = 0.967530
13	70%C, 30%F-6	144	142	147	147	145.0	
14	70%C, 30%F-7	152	152	146	146	149.0	
15	85%C, 15%F-1	92	93	93	93	92.8	
16	85%C, 15%F-2	102	102	105	105	103.5	
17	85%C, 15%F-3	119	119	116	116	117.5	a = 0.007501
18	85%C, 15%F-4	129	129	123	123	126.0	b = 0.660015
19	85%C, 15%F-5	141	141	141	141	141.0	R^2^ = 0.953415
20	85%C, 15%F-6	127	128	145	145	136.3	
21	85%C, 15%F-7	155	155	150	151	152.8	
22	55%C, 45%F-1	78	79	77	78	78.0	
23	55%C, 45%F-2	89	89	88	87	88.3	
24	55%C, 45%F-3	94	94	98	98	96.0	
25	55%C, 45%F-4	120	121	115	114	117.5	a = 0.006733
26	55%C, 45%F-5	133	132	116	116	124.3	b = 0.636062
27	55%C, 45%F-6	123	123	132	132	127.5	R^2^ = 0.980954
28	55%C, 45%F-7	144	144	150	151	147.3	
29	55%C, 45%F-8	144	145	162	162	153.3	
30	55%C, 45%F-9	155	150	166	168	159.8	
31	0%C, 100%F-1	105	106	105	105	105.3	
32	0%C, 100%F-2	115	115	123	125	119.5	
33	0%C, 100%F-3	125	125	133	134	129.3	a = 0.007514
34	0%C, 100%F-4	145	145	145	146	145.3	b = 0.329800
35	0%C, 100%F-5	141	142	148	149	145.0	R^2^ = 0.919727
36	0%C, 100%F-6	144	144	148	148	146.0	
37	0%C, 100%F-7	154	155	165	165	159.8	
38	100%C+, 0%F-1	93	92	-	-	92.5	
39	100%C+, 0%F-2	102	102	-	-	102.0	a = 0.008734
40	100%C+, 0%F-3	114	113	-	-	113.5	b = 0.954350
41	100%C+, 0%F-4	141	139	-	-	140.0	R^2^ = 0.963781
42	100%C+, 0%F-5	158	160	-	-	159.0	
43	80%C, 20%C+, 0%F-1	97	98	-	-	97.5	
44	80%C, 20%C+, 0%F-2	114	114	-	-	114.0	a = 0.008849
45	80%C, 20%C+, 0%F-3	133	135	-	-	134.0	b = 0.683382
46	80%C, 20%C+, 0%F-4	156	156	-	-	156.0	R^2^ = 0.977411
47	80%C, 20%C+, 0%F-5	161	163	-	-	162.0	

**Table 5:** Results from the mixtures with shrinkage-reducing admixture.

#	Mixture	Sp/p	D0’1a	D0’1b	D0’2a	D0’2b	D0	t0	D60’1a	D60’1b	D60’2a	D60’2b	D60	t60
48	100%C, SpA1.0%	1.0%	74	73	-	-	73.5	-	-	-	-	-	-	-
49	100%C, SpA1.5%	1.5%	133	134	-	-	133.5	-	-	-	-	-	-	-
50	100%C, SpA2.0%	2.0%	180	183	-	-	181.5	-	-	-	-	-	-	-
51	100%C, SpA2.5%	2.5%	266	218	-	-	242.0	-	-	-	-	-	-	-
52	100%C, SpB1.0%	1.0%	216	214	-	-	215.0	-	-	-	-	-	-	-
53	100%C, SpB2.0%	2.0%	237	242	-	-	239.5	-	-	-	-	-	-	-
54	100%C, SpB2.6%	2.6%	236	242	-	-	239.0	-	-	-	-	-	-	-
55	w/c.18, SpC1.0%	1.0%	-	-	-	-	-	336	-	-	-	-	-	-
56	w/c.18, SpC1.5%	1.5%	-	-	-	-	-	205.72	-	-	-	-	-	-
57	w/c.18, SpC2.0%	2.0%	-	-	-	-	-	194.42	-	-	-	-	-	-
58	w/c.18, SpC3.0%	3.0%	-	-	-	-	-	188.69	-	-	-	-	-	-
59	w/c.18, SpC1.3%	1.3%	-	-	-	-	-	232.43	-	-	-	-	-	-
60	w/c.18, SpC1.7%	1.7%	-	-	-	-	-	184.52	-	-	-	-	-	-
61	w/c.225, SpC0.5%	0.5%	-	-	-	-	-	116.6	-	-	-	-	-	-
62	w/c.225, SpC0.8%	0.8%	-	-	-	-	-	89.5	-	-	-	-	-	-
63	w/c.225, SpC1.2%	1.2%	-	-	-	-	-	82.7	-	-	-	-	-	-
64	w/c.225, SpC1.5%	1.5%	-	-	-	-	-	91.56	-	-	-	-	-	-
65	w/c.225, SpC1.0%	1.0%	-	-	-	-	-	87.72	-	-	-	-	-	-
66	w/c.225, SpC0.6%	0.6%	-	-	-	-	-	98.41	-	-	-	-	-	-
67	w/c.225, SpC0.7%	0.7%	-	-	-	-	-	94.68	-	-	-	-	-	-
68	w/c.225, SpC0.9%	0.9%	-	-	-	-	-	92.69	-	-	-	-	-	-
69	w/c.225, SpC1.1%	1.1%	-	-	-	-	-	93.91	-	-	-	-	-	-
70	w/c.225, SpC1.35%	1.35%	-	-	-	-	-	86.22	-	-	-	-	-	-
71	63%C, 37%F, SpC0.4%	0.4%	126	122	131	122	125.3	119.78	-	-	-	-	-	-
72	63%C, 37%F, SpC0.5%	0.5%	193	191	196	194	193.5	64.03	-	-	-	-	-	-
73	63%C, 37%F, SpC0.6%	0.6%	206	198	198	200	200.5	59.09	191	191	183	192	189.3	106.81
74	63%C, 37%F, SpC0.7%	0.7%	201	206	201	208	204.0	56.00	203	201	202	201	201.8	85.69
75	63%C, 37%F, SpC0.8%	0.8%	217	218	217	213	216.3	54.12	206	204	206	205	205.3	68.50
76	63%C, 37%F, SpC0.9%	0.9%	211	202	200	206	204.8	54.78	211	204	211	202	207.0	66.34
77	63%C, 37%F, SpC1.0%	1.0%	203	217	203	217	210.0	55.47	210	216	211	214	212.8	67.13
78	63%C, 37%F, SpC1.1%	1.1%	207	206	208	206	206.8	57.59	202	197	202	201	200.5	64.59
79	63%C, 37%F, SpD0.5%	0.5%	187	187	185	177	184.0	79.57	-	-	-	-	-	-
80	63%C, 37%F, SpD0.6%	0.6%	200	203	200	198	200.3	69.90	-	-	-	-	-	-
81	63%C, 37%F, SpD0.7%	0.7%	204	206	206	209	206.3	67.59	176	181	171	171	174.8	189.72
82	63%C, 37%F, SpD0.8%	0.8%	205	204	198	202	202.3	65.81	188	177	191	185	185.3	129.63
83	63%C, 37%F, SpD0.9%	0.9%	210	200	206	209	206.3	65.03	201	195	201	197	198.5	112.90
84	63%C, 37%F, SpD01.0%	1.0%	206	196	209	205	204.0	69.97	198	195	202	187	195.5	101.72
85	63%C, 37%F, SpD01.1%	1.1%	198	202	198	198	199.0	63.71	197	198	194	200	197.3	98.87
86	63%C, 37%F, SpD01.2%	1.2%	206	206	206	197	203.8	70.1	198	197	190	195	195.0	108.20
87	63%C, 37%F, SpD01.3%	1.3%	204	205	196	200	201.3	75.53	194	192	193	190	192.3	112.70
88	63%C, 37%F, SpD01.4%	1.4%	192	195	190	193	192.5	72.6	195	192	193	190	192.5	106.90
82	Ref.	0.8%	205	204	198	202	202.3	65.81	188	177	191	185	185.3	129.63
A	SRA20, wr0%	0.8%	212	220	221	221	218.5	57.00	212	212	204	194	205.5	81.00
B	SRA20, wr100%	0.8%	214	214	218	215	215.3	67.69	209	215	208	205	209.3	93.54
C	SRA20, wr50%	0.8%	215	215	210	215	213.8	66.38	209	209	207	208	208.3	90.44
D	SRA40, wr50%	0.8%	208	208	214	210	210.0	65.22	204	205	202	204	203.8	88.50

## Data Availability

Not applicable.
